# Evaluation of the Potential of Modified Calcium Carbonate as a Carrier for Unsaturated Fatty Acids in Oxygen Scavenging Applications

**DOI:** 10.3390/ma14175000

**Published:** 2021-09-01

**Authors:** Bettina Röcker, Gabriel Mäder, Fabien Wilhelm Monnard, Magdalena Jancikova, Matthias Welker, Joachim Schoelkopf, Selçuk Yildirim

**Affiliations:** 1 Institute of Food and Beverage Innovation, Department of Life Sciences and Facility Management, Zurich University of Applied Sciences, Campus Reidbach, 8820 Wädenswil, Switzerland; bettina.roecker@zhaw.ch (B.R.); gabriel.maeder@strombriefe.ch (G.M.); magdalena.jancikova@zhaw.ch (M.J.); 2 Omya International AG, Baslerstrasse, 4665 Oftringen, Switzerland; fabien.monnard@omya.com (F.W.M.); matthias.welker@omya.com (M.W.); joachim.schoelkopf@omya.com (J.S.)

**Keywords:** active packaging, oxygen scavenger, unsaturated fatty acids, linoleic acid, oleic acid, modified calcium carbonate

## Abstract

Modified calcium carbonates (MCC) are inorganic mineral-based particles with a large surface area, which is enlarged by their porous internal structure consisting of hydroxyapatite and calcium carbonate crystal structures. Such materials have high potential for use as carriers for active substances such as oxygen scavenging agents. Oxygen scavengers are applied to packaging to preserve the quality of oxygen-sensitive products. This study investigated the potential of MCC as a novel carrier system for unsaturated fatty acids (UFAs), with the intention of developing an oxygen scavenger. Linoleic acid (LA) and oleic acid (OA) were loaded on MCC powder, and the loaded MCC particles were characterized and studied for their oxygen scavenging activity. For both LA and OA, amounts of 20 wt% loading on MCC were found to provide optimal surface area/volume ratios. Spreading UFAs over large surface areas of 31.6 and 49 m^2^ g^−1^ MCC enabled oxygen exposure and action on a multitude of molecular sites, resulting in oxygen scavenging rates of 12.2 ± 0.6 and 1.7 ± 0.2 mL O_2_ d^−1^ g^−1^, and maximum oxygen absorption capacities of >195.6 ± 13.5 and >165.0 ± 2.0 mL g^−1^, respectively. Oxygen scavenging activity decreased with increasing humidity (37–100% RH) and increased with rising temperatures (5–30 °C). Overall, highly porous MCC was concluded to be a suitable UFA carrier for oxygen scavenging applications in food packaging.

## 1. Introduction

Oxygen can negatively affect the quality of packaged food, as it can promote oxidative or microbial spoilage of the food product. This can lead to color change, loss of nutritional value or sensorial alteration of the food due to the formation of by-products, which can produce off flavours and undesired odours [1,2]. To overcome this, oxygen sensitive products are mainly packed by flushing the headspace with nitrogen or applying modified atmosphere, replacing the air in the headspace with a defined gas mixture without oxygen. However, it is not possible to fully remove the oxygen, due to inefficiencies during the flushing or evacuation process. Additionally, oxygen may also diffuse through the packaging material while it is being stored, which may still result in a decrease in food quality. Oxygen scavengers, however, have the potential to remove the residual oxygen from a package, as well as any oxygen that diffuses through the packaging material at a later time [3,4]. Although several oxygen scavengers have been developed and characterized [4,5], most commercially available oxygen scavengers are iron-based sachets [6]. Sachet-based applications, however, have several drawbacks. For instance, the sachets could accidentally rupture, leading to involuntary consumption. In addition, they require an additional packaging step, and they lack consumer acceptance [7,8], especially when they are used with certain foods like meat products [7,9,10]. Thus, active ingredients should be preferably integrated into the packaging materials. Some of the oxygen scavenging agents, such as iron [11,12,13] or palladium [14,15,16], have been successfully incorporated into the packaging films by using extrusion, impregnation, or vacuum coating technologies. However, integrating other scavenging agents (e.g., gallic acid, ascorbic acid, enzymes, and unsaturated fatty acids) remains challenging [17].

With respect to matrix integration, highly porous particles are increasingly being used as carriers for active ingredients [18,19] for pharmaceutical and food applications, to increase solubility of hydrophobic substances in water [20] or to control the release of active ingredients [21,22,23]. Omya International AG has developed a range of modified calcium carbonates (MCC), small inorganic composite particles (5–15 µm) of hydroxyapatite and calcium carbonate crystals with a hydrophilic surface and high porosity and surface area [24]. This development is based on exposing natural ground calcium carbonate to acids under specific conditions. Their high surface area, which is enlarged by their porous internal structure, makes them potential candidates for use as carriers for oxygen scavenging agents. Thereby, the extended surface area of MCC is ideal for higher oxidation kinetics [25]. In a recent study, MCC has been shown to have high potential to dope liquid agents such as essential oils [23] and for integration into the packaging as a coating. Therefore, MCC is believed to have a high potential for use as a carrier system for oxygen scavenging agents being integrated into packaging.

Unsaturated fatty acids (UFAs) are commonly used as plasticizers for the production of packaging materials [26,27,28], but they are also known for their ability to scavenge oxygen [29,30,31]. Thereby, the scavenging mechanism relies on autoxidation reactions with atmospheric triplet oxygen (^3^O_2_) and, in contrast to some other scavenging systems, it does not require the presence of water for propagation of the reaction [2,29]. Thus, UFA-based oxygen scavenging systems have great potential for use as oxygen scavengers, especially for dry products [32]. In the present study, the potential of MCC was therefore investigated as a novel carrier system for UFAs. In this context, MCC has been utilised for oxygen scavenging purposes for the first time. Oleic acid (OA, octadecenoic acid, 18:1) and linoleic acid (LA, octadecadienoic acid, 18:2) were loaded on the MCC powder and their oxygen scavenging potential was evaluated. In particular, the effects of the UFA loading amount, temperature, and humidity on the oxygen scavenging activity were investigated. In addition, the oxygen scavenging capacity was determined and the storage stability of the MCC loaded with oleic and linoleic acid was evaluated.

## 2. Materials and Methods

### 2.1. Loading of MCC with UFAs

Modified calcium carbonates (MCC) were supplied by Omya International AG (Oftringen, Switzerland) as a powder of median particle size (Malvern Mastersizer 3000, Almelo, The Netherlands; dry measurements, d50%) of 5.0 µm, top cut (d98%) of 13 µm and specific surface area [33] of 103 m^2^ g^−1^. Oleic acid (OA, 364525, technical grade 90%) and linoleic acid (LA, 62240, technical 60–74%, GC) were supplied by Sigma-Aldrich, Buchs SG, Switzerland.

MCC was loaded with 9.1, 20, and 41.2 wt% oleic acid and 10, 20, and 30 wt% linoleic acid. For loading on the MCC, the unsaturated fatty acids (UFAs) were added dropwise to the MCC powder under constant mechanical stirring (300 rpm) using a lab stirrer (Somakon MP-GL, Somakon Verfahrenstechnik UG, Germany; vessel volume: 2.5 L) at room temperature. The loaded MCC was stirred for 10 additional minutes after adding the whole amount of the respective UFA. The loaded MCC was stored under nitrogen at 21 ± 1 °C in the dark until use.

### 2.2. Characterisation of MCC Loaded with UFAs

#### 2.2.1. Mercury Intrusion Porosimetry (MIP)

The specific pore volume was measured using a mercury intrusion porosimetry measurement using a Micromeritics Autopore V 9620 mercury porosimeter with a maximum applied pressure of mercury 414 MPa (60,000 psi), equivalent to a Laplace throat diameter of 0.004 µm. The equilibration time used at each pressure step was 20 s. The sample material was sealed in a 5 mL chamber powder penetrometer for analysis. The data were corrected for mercury compression, penetrometer expansion, and sample material compression according to the methodology presented in the study of Gane [34] using the software “Pore-Cor” (pore-level properties correlator).

#### 2.2.2. Brunauer–Emmett–Teller (BET) Analysis of the Specific Surface Area

**Figure A1:**
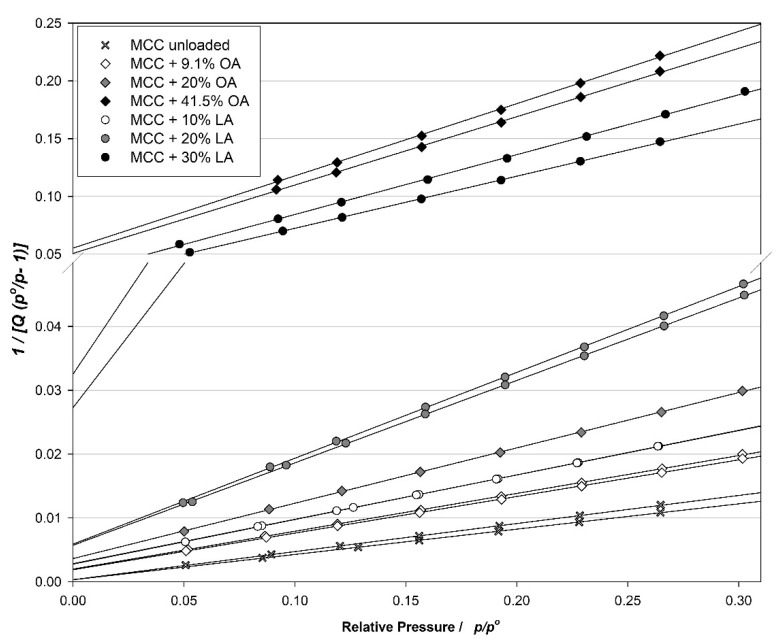
BET Surface area plots of loaded and unloaded modified calcium carbonates (MCC) (n = 2, Q = N_2_ amount adsorbed m^3^ g^−1^).

The specific surface area was determined using the Brunauer–Emmett–Teller (BET) standard method (ISO 9277–2010 [33]). Nitrogen adsorption was recorded at 77.35 K using a Micromeritics TriStar II Plus surface area analyser (ATS Scientific Inc., Burlington, ON, Canada). The surface area was specified in m^2^ g^−1^ by the MicroActive software TriStar II 3020 Version 3.02 according to the BET surface area plots shown in Figure A1 in the Appendix A.

#### 2.2.3. Field Emission Scanning Electron Microscopy (SEM)

MCC powder was immobilised on C-Tape and sputtered with a gold layer of 8 nm (Safematic CCU-010 HV, Zizers, Switzerland). Samples were analysed with field-emission scanning electron microscope (FESEM, Zeiss Sigma VP, Oberkochen, Germany; acceleration voltage: 2 kV, aperture: 30 μm, distance: 5 mm, detector: secondary electron detector SE2).

### 2.3. Sample Preparation for Evaluation of Oxygen Scavenging Activity

Sample preparation was performed in a clove box (CaptairPyramid, Erlab, Val-de-Reuil, France) under exclusion of oxygen (about 0.1 vol.-%). Therefore, 1.000 ± 0.010 g of MCC loaded with UFA or pure MCC (negative control) were filled in heat sealable tea bags (55 mm × 80 mm, Special Tea Company^®^, Orlando, FL, USA) and sealed with a hand heat impulse sealer (400 HC, TRL EMC Ltd., Skelmersdale, UK). Pure oleic and linoleic acid were also tested, in the same amount as what was loaded on MCC. For that purpose, the pure UFA were placed in a glass petri dish lid (9.5 cm inner diameter, 1 cm height). Sample preparation was performed just prior to packaging.

### 2.4. Setting of Controlled in-Package Humidity

Control of the relative humidity (RH) in the packaging headspace was accomplished using different saturated salt solutions resulting in RH’s of 37, 47, 67, 77, 83, 87, 100% [35,36,37]. Magnesium chloride (anhydrous > 98.0%), potassium carbonate (BioXtra, ≥99.0%), sodium hydrogen sulphate (technical grade), sodium chloride (BioXtra, ≥99.5%) ammonium sulphate (BioUltra, ≥99.0%), sodium sulphate (anhydrous, ≥99.0%), and demineralized water, were applied. All salts were supplied by Sigma-Aldrich, Buchs SG, Switzerland. Each salt solution was prepared in the same way by dissolving the salt in demineralized water via strong shaking and placing in an ultrasonic bath (USC1200D, VWR International, Leuven, Belgium) for 10 min at 25 °C on power-level 9. Solutions of salts with exothermic enthalpy were periodically placed in an ice water bath to accelerate the solution process. To ensure saturation of the solutions, salt was added in excess. All solutions were degassed in the ultrasonic bath for 5 min, degassed with nitrogen, and stored at 21 °C in the dark. To inhibit microbial growth in the pure demineralized water, silver nitrate (Silver nitrate solution, 0.1 N, Merck KGaA, Darmstadt, Germany) was added in a concentration of 0.2 mmol L^−1^. For packaging, 20 mL of each solution was placed in a glass petri dish lid (9.5 cm inner diameter, 1 cm height). To ensure that the solutions remained saturated during the measurements, approximately 1 g of the corresponding salts was added to the solution.

### 2.5. Packaging Process and Measurement of Oxygen Scavenging Activity

**Figure 1:**
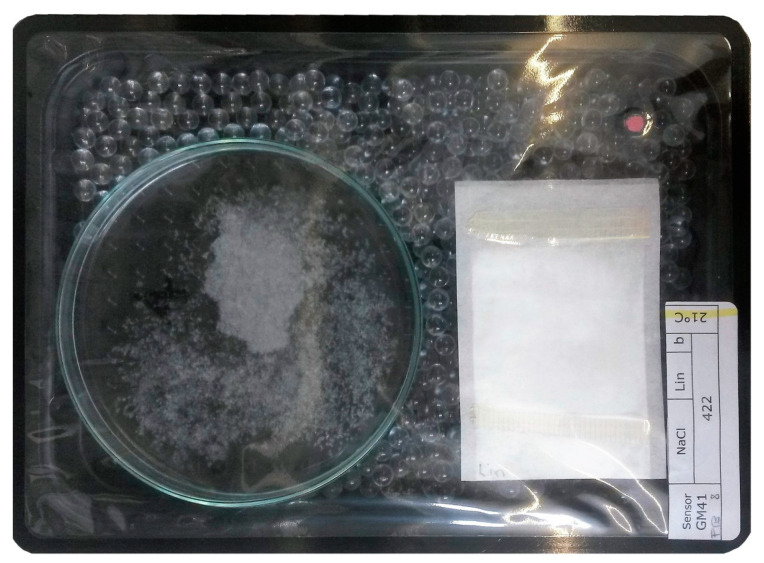
Example package setting for oxygen scavenging measurements. High barrier tray containing 1 g modified calcium carbonate (MCC) powder loaded with UFAs in a sealable teabag fixed on the lidding film, a petri dish filled with a saturated salt solution, glass beads to adjust the headspace volume and an oxygen sensitive sensor spot (red).

High barrier packaging trays (PS-EVOH-PE with peel, 0.5 mm, 204 mm× 147 mm, 8 and 14 mm height, Stäger & Co AG, Muri, Switzerland) were used. For packages containing tea bags only, trays which were 8 mm in height were used (volume 250 mL). For the tests under controlled RH and pure UFAs, the petri dish containing the water or salt solution was placed in trays which were 14 mm in height and glass beads were added to ensure a uniform headspace volume of 250 mL for all experiments. Tea bags filled with MCC or loaded MCC were fixed on the inner side of the lidding film (Ecoweb M/Pap 57 AF, PET/PE/EVOH/PE peel, Südpack Verpackungen, Ochsenhausen, Germany; O_2_ transmission rate ≤ 2.5 cm^3^ m^−2^ d^−1^ bar^−1^ at 23 °C and 50% relative humidity) with an adhesive tape (tesafix^®^ 4934, Tesa, Offenburg, Germany). To evaluate the oxygen scavenging activity (OSA), an oxygen sensitive sensor spot (PST3 for normal atmosphere (NA) and PST6 for modified atmosphere (MAP), 3 mm diameter, PreSens, Regensburg, Germany) was glued to the inner side of the lidding film prior to sealing. Figure 1 shows an example package setting.

The NA packages were sealed at 125 °C using a tray sealer (T200, Multivac, Hünenberg, Switzerland). For MAP, a vacuum of 50 mbar was applied, the tray was flushed with a gas mixture (2 vol.-% O_2_ and 98 vol.-% N_2_) until 900 mbar was achieved and then sealed at 125 °C. The OSA measurements were performed by a non-destructive measurement method using fibre optic optodes Fibox 4 trace (PreSens, Regensburg, Germany). The measurements were carried out in a climate chamber (KBF 720, Binder GmbH, Tuttlingen, Germany) at 21 ± 1 °C and 30 ± 1 °C at 50% RH and in a cold-storage room (Kolb Kälte AG, Rüthi, Switzerland) at 5 ± 1 °C. To simplify, oxygen scavenging rates (OSR) were calculated by determining the slope of the initial linear part of the progression of oxygen concentration in the package. All experiments were carried out in triplicate or quadruplicate.

## 3. Results

Surface-modified calcium carbonate (MCC) powder was loaded with unsaturated fatty acids (UFAs), namely oleic acid (OA) and linoleic acid (LA), with the intention to develop novel oxygen scavenging applications. To evaluate the potential of MCC as a carrier for UFAs, structural properties of MCC loaded with UFAs were analysed and their oxygen scavenging performance was evaluated under varying conditions.

### 3.1. Structural Properties of MCC Loaded with UFAs

**Figure 2:**
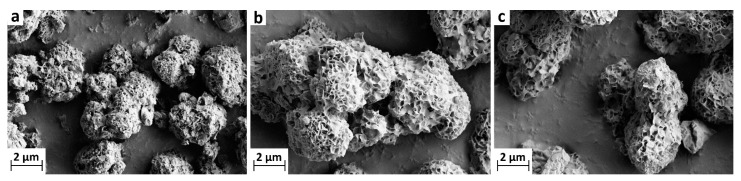
Scanning electron microscope (SEM) images of modified calcium carbonate (MCC) at 7500× magnification: (**a**) unloaded MCC, (**b**) MCC loaded with linoleic acid (20 wt%) and (**c**) MCC loaded with oleic acid (20 wt%).

MCC and MCC loaded with LA and OA were characterized with respect to their intra particle pore volume (Mercury intrusion porosimetry) and specific surface area (Brunauer–Emmett–Teller (BET) analysis). BET surface area plots are presented in Figure A1 in the Appendix A and the specific surface areas are listed in Table 1. The results clearly demonstrate that the void space of the unloaded MCC was partially filled with UFAs. The increase in the loading amount of UFAs (from 9.1–41.5 wt% for OA and 10–30 wt% for LA) resulted in a decrease in the intra particle specific pore volume (from 0.802–0.163 cm^−3^ g^−1^ for OA and from 0.766–0.352 cm^−3^ g^−1^ for LA) as well as in the specific surface area (from 72–6.6 m^2^ g^−1^ for OA and from 60–8.9 m^2^ g^−1^ for LA). The results also show that even with the highest loading amounts of UFA, the pores of the MCCs have not been fully filled. This was also confirmed by the scanning electron microscopy (SEM) images of the MCC (Figure 2a) and MCC loaded with UFA (Figure 2b,c). As can be seen, the porous structure of the MCC loaded with 20 wt% LA (Figure 2b) or 20 wt% of OA (Figure 2c) was retained and the pores were not fully loaded.

### 3.2. Oxygen Scavenging Activity

The oxygen scavenging activity of MCC loaded with UFAs was evaluated by monitoring the decrease in the oxygen concentration in the headspace of the high barrier trays (containing loaded MCC) over time. An initial oxygen concentration of 2 vol.-% was introduced into the package to imitate the residual amount of oxygen remaining in packages at industrial production lines. The effect of the loading amount of UFAs, as well as the effects of temperature and relative humidity on the oxygen scavenging activity of the loaded MCCs were studied. Additionally, maximum oxygen absorption capacity and storage stability of the loaded MCC was evaluated.

#### 3.2.1. Effect of Loading Amount on the Oxygen Scavenging Activity of MCC Loaded with UFAs

**Figure 3:**
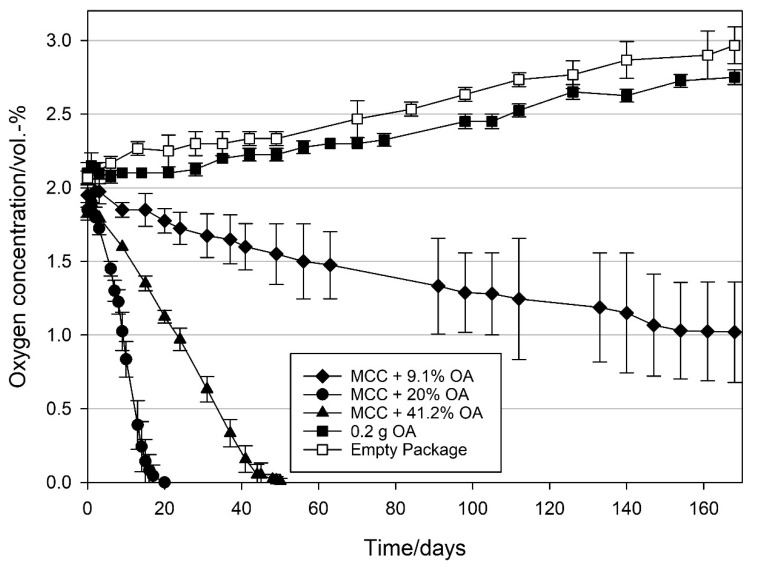
Oxygen concentration in packages containing 1 g modified calcium carbonate (MCC) loaded with 9.1, 20, and 41.2 wt% oleic acid, 0.2 g pure oleic acid and empty package. Packed under modified atmosphere (MAP, 2 vol.-% O_2_, rest N_2_; headspace 250 mL) and stored at 21 °C. Mean values and standard deviation (n = 4).

In order to find the optimal loading amount of oleic and linoleic acid, three different amounts of each UFA were loaded on MCC and the oxygen scavenging activity (OSA) of the loaded MCC was evaluated. For comparison, the OSA of pure oleic and linoleic acid, was measured under the same conditions. As shown in Figure 3, with a loading amount of 20 wt% oleic acid (OA), the 2 vol.-% initial headspace oxygen was removed within 20 days resulting in an oxygen scavenging rate of (OSR) of 1.7 ± 0.2 mL O_2_ d^−1^ g_OA_^−1^. For both higher (41.2 wt% OA) and lower (9.1 wt% OA) loading amounts, OSRs were remarkably lower with 0.3 ± 0.0 and 0.2 ± 0.1 mL O_2_ d^−1^ g_OA_^−1^, respectively. Whereas with the MCC loaded with the 41.2 wt% OA 50 days were required to remove all the headspace oxygen, with the 9.1 wt% loading, and it was not possible to remove the 2 vol.-% oxygen within the six month-test period. In order to compare the oxygen scavenging activity of the loaded MCC with the pure OA, 0.2 g pure OA (referring to the OA amount in the 20 wt% sample) was packed and stored under the same conditions. As can be seen in Figure 3, the evolution of oxygen concentration in the package with pure OA is very similar to the empty packages, indicating that the pure OA alone was not able to scavenge any significant amount of oxygen within the test period.

**Figure 4:**
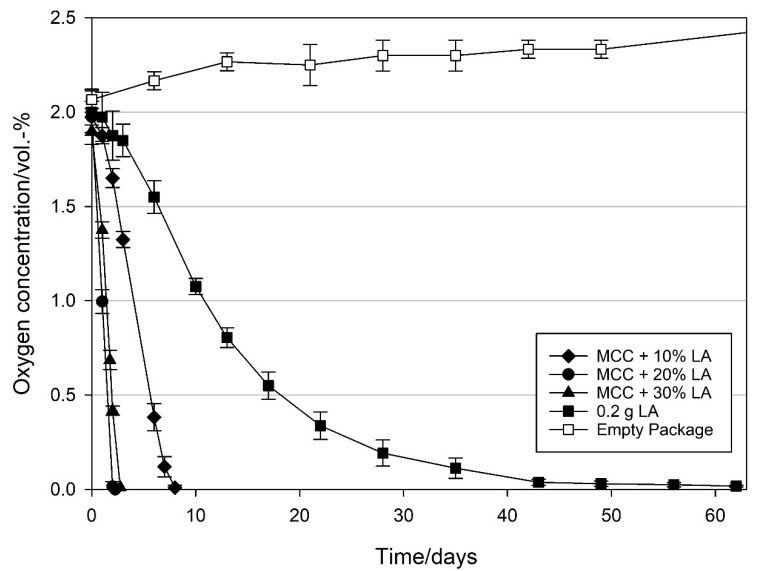
Oxygen concentration in packages containing 1 g modified calcium carbonate (MCC) loaded with 10, 20, and 30 wt% linoleic acid, 0.2 g pure linoleic acid and empty package. Packed under modified atmosphere (MAP, 2 vol.-% O_2_, rest N_2_; headspace 250 mL) and stored at 21 °C. Mean values and standard deviation (n = 4).

When MCC was loaded with linoleic acid (LA), OSA was remarkably higher for all loading amounts (10, 20, and 30 wt% LA) compared to that of OA. As shown in Figure 4, with a loading amount of 10 wt% LA, the 2 vol.-% initial headspace oxygen was removed within eight days with an OSR of 7.8 ± 0.4 mL O_2_ d^−1^ g_LA_^−1^. When the loading amount of LA was increased to 20 wt%, time to scavenge all oxygen in the package decreased to two days resulting in an OSR of 12.2 ± 0.6 mL O_2_ d^−1^ g_LA_^−1^. Further increase of the loading amount to 30 wt% resulted in a decrease in oxygen scavenging activity (6.5 ± 0.6 mL O_2_ d^−1^ g_LA_^−1^) and the time to remove all oxygen in the headspace increased to 2.75 days. In contrast to pure OA (Figure 4), pure LA showed oxygen scavenging activity. By applying 0.2 g of pure LA (referring to the LA amount in the 20 wt% sample), the 2 vol.-% initial headspace oxygen was removed within 43 days resulting in an OSR of 1.4 ± 0.1 mL O_2_ d^−1^ g_LA_^−1^.

#### 3.2.2. Effect of Temperature on the Oxygen Scavenging Activity of MCC Loaded with UFAs

**Figure 5:**
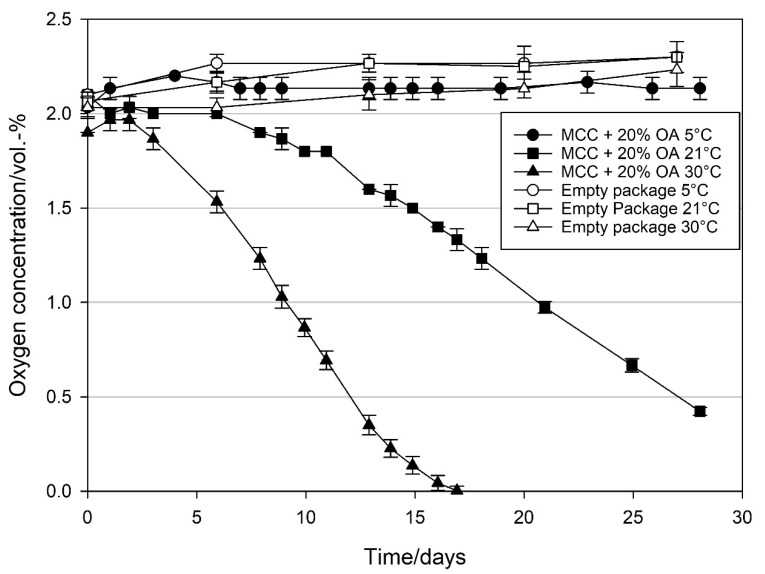
Oxygen concentration in packages containing 1 g modified calcium carbonate (MCC) loaded with 20 wt% oleic acid stored at 5, 21, and 30 °C, and ~50% RH inside the package and of empty package settings stored under the same conditions. Packed under modified atmosphere (MAP, 2 vol.-% O_2_, rest N_2_; headspace 250 mL). Mean values and standard deviation (n = 3).

**Figure 6:**
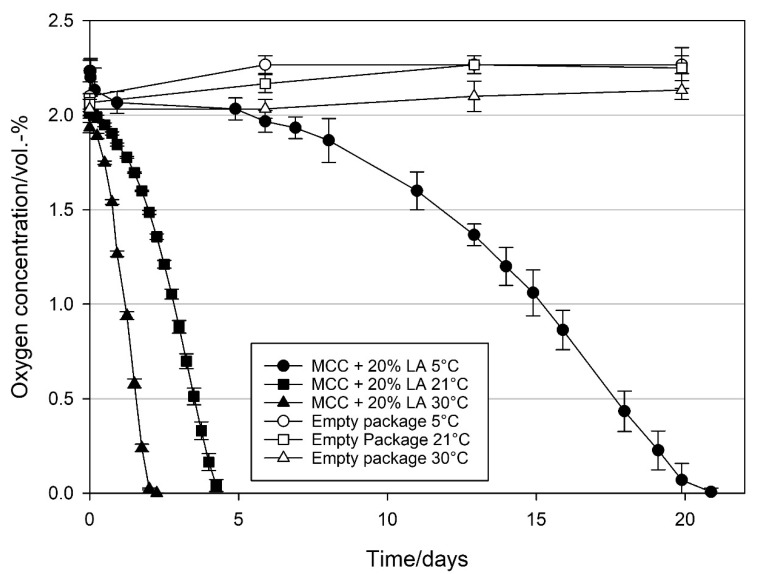
Oxygen concentration in packages containing 1 g modified calcium carbonate (MCC) loaded with 20 wt% linoleic acid stored at 5, 21, and 30 °C, and ~50% RH in the package and of empty packages stored under the same conditions. Packed under modified atmosphere (MAP, 2 vol.-% O_2_, rest N_2_; headspace 250 mL). Mean values and standard deviation (n = 3).

To examine the effect of temperature on the oxygen scavenging activity of loaded MCC, MCC was loaded with 20 wt% oleic acid and linoleic acid, and their oxygen scavenging activities were studied at 5, 21, and 30 °C. The relative humidity in the package was about 50% RH. Figure 5 and Figure 6 clearly show that temperature has a strong impact on the oxygen scavenging activity of MCC loaded with UFAs. When MCC loaded with OA were stored at 30 °C, the initiation phase of the oxidation, where the oxygen concentration did not decrease significantly, was only two days. Afterwards, in the propagation phase, oxygen concentration started to decrease and reached <0.01 vol.-% between 16 and 17 days, resulting in an OSR of 2.1 ± 0.0 mL O_2_ d^−1^ g_OA_^−1^ (determined between day 3 and 15). Decrease in temperature to 21 °C increased the initiation phase to six days. Afterwards oxygen concentration decreased to 0.42 vol.-% within 28 days with a lower OSR of 1.0 ± 0.0 mL O_2_ d^−1^ g_OA_^−1^ (determined between day 6 and 28). When the temperature was further decreased to 5 °C, the initiation phase was further extended to more than 28 days and no significant decrease in the oxygen concentration was observed during this period.

When MCC loaded with LA was stored at 30 °C, oxygen concentration started to decrease immediately and reached <0.01 vol.-% after two days, resulting in an OSR of 16.6 ± 0.1 mL O_2_ d^−1^ g_LA_^−1^ (Figure 6). Decrease in temperature to 21 °C reduced the OSR to 8.2 ± 0.2 mL O_2_ d^−1^ g_LA_^−1^ and all the oxygen was scavenged within 4.5 days. At both conditions, no clear initiation phase was observed. Conversely, at 5 °C, an initiation phase was observed to last five days. Afterwards, oxygen concentration decreased to <0.01 vol.-% after 21 days resulting in an OSR of 2.1 ± 0.2 mL O_2_ d^−1^ g_LA_^−1^.

#### 3.2.3. Effect of Relative Humidity on the Oxygen Scavenging Activity of MCC Loaded with UFAs

**Figure 7:**
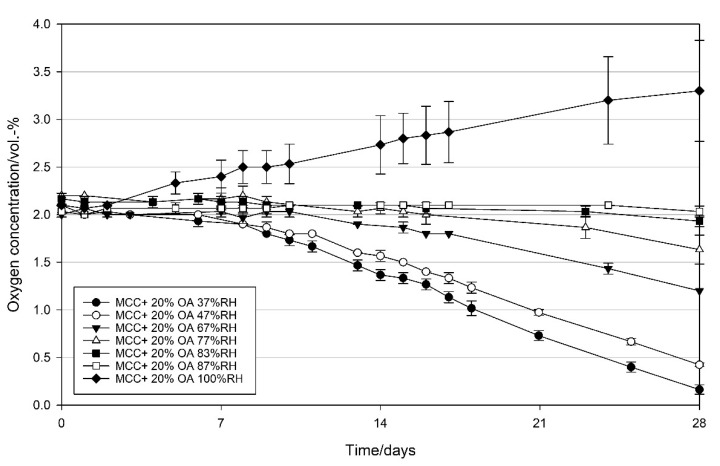
Oxygen concentration in packages containing 1 g modified calcium carbonate (MCC) loaded with 20 wt% oleic acid stored at 21 °C, and 37–100% RH in the package. Packed under modified atmosphere (MAP, 2 vol.-% O_2_, rest N_2_; headspace 250 mL). Mean values and standard deviation (n = 3).

**Figure A2:**
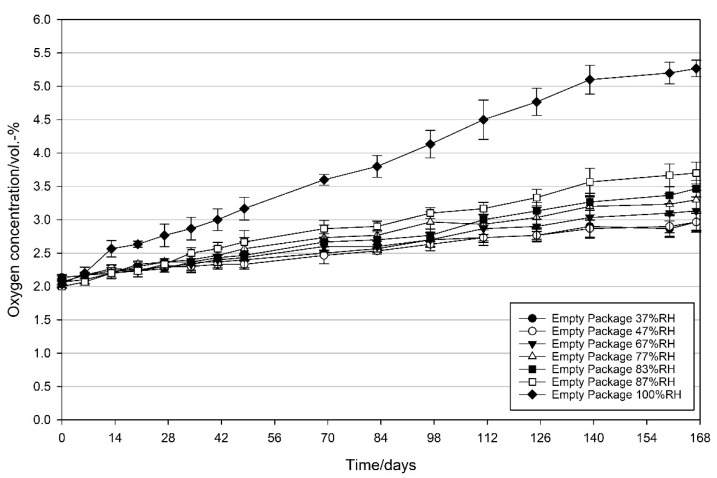
Oxygen concentration in empty packages stored at 21 °C, and 37–100% RH in the package. Packed under modified atmosphere (MAP, 2 vol.-% O_2_, rest N_2_; headspace 250 mL). Mean values and standard deviation (n = 3).

Oleic acid and linoleic acid loaded MCC was packed under various relative humidities inside the packages, ranging from 37–100% RH and their oxygen scavenging activity was evaluated. Figure 7 shows the effect of relative humidity on the oxygen scavenging activity (OSA) of OA loaded MCC. As can be seen in Figure 7, relative humidity negatively affects the oxygen scavenging activity of the loaded MCC. Under relatively dry conditions (37 and 47% RH), an initiation phase of about six days was observed. Afterwards, an oxygen scavenging rate of 1.1 ± 0.0 mL O_2_ d^−1^ g_OA_^−1^ at both 37 and 47% RH was observed. At 67% RH, the initial phase was prolonged to 10 days, and the OSR was decreased to 0.6 ± 0.0 mL O_2_ d^−1^ g_OA_^−1^. At higher relative humidities, the end of the initial phase could not be recognized during the measurement time of 28 days. At 87 and 100% RH, no OSA was observed. In the packages with 83 and 87% RH, the oxygen concentration remained relatively constant, while in the packages with 100% RH it increased from an initial value of 2.1–3.3 vol.-% within 28 days. This can be attributed to the oxygen ingress from the environment as shown in Figure A2 in the Appendix A. Packaging materials (tray and lidding film) used in this study contained ethylene vinyl alcohol (EVOH) as an oxygen barrier. At high relative humidity, the permeability of EVOH for oxygen increases [38]. In empty packages with humidities from 37–87% RH, only a slight increase in oxygen concentration of 0.3 ± 0.1 vol.-% was detected within the 28 days, while under 100% RH an oxygen ingress of 0.7 ± 0.2 vol.-% was observed (Figure A2).

**Figure 8:**
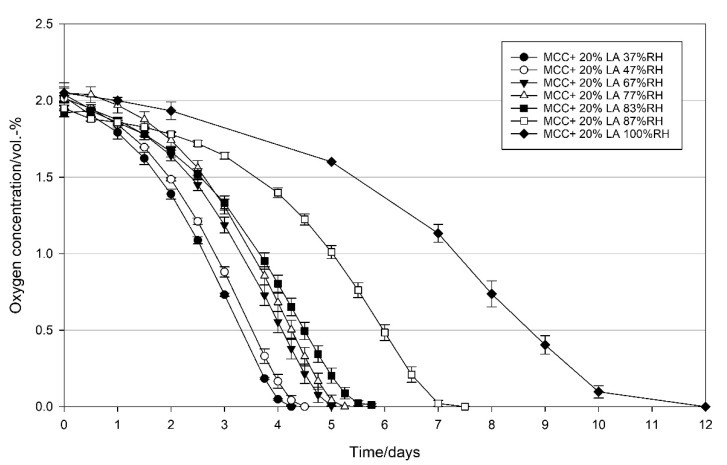
Oxygen concentration in packages containing 1 g modified calcium carbonate (MCC) loaded with 20 wt% linoleic acid stored at 21 °C, and 37–100% RH in the package. Packed under modified atmosphere (MAP, 2 vol.-% O_2_, rest N_2_; headspace 250 mL). Mean values and standard deviation (n = 3).

A similar effect of relative humidity on the oxygen scavenging activities, i.e., decrease in the oxygen scavenging activity accompanied by an increase in relative humidity was observed for the linoleic acid loaded MCC (Figure 8). LA loaded MCC, however, could successfully scavenge all the oxygen in the headspace within 12 days, with 4.25 days being the fastest at 37% RH, and 12 days being required at 100% RH. With regard to the OSA of LA loaded MCC at 100% RH, shown in Figure 8, an oxygen ingress of 0.5 vol.-% has to be considered for day 12, as shown in Figure A2, while for lower RHs this ingress is negligible.

### 3.3. Oxygen Absorption Capacity of MCC Loaded with UFAs

**Figure 9:**
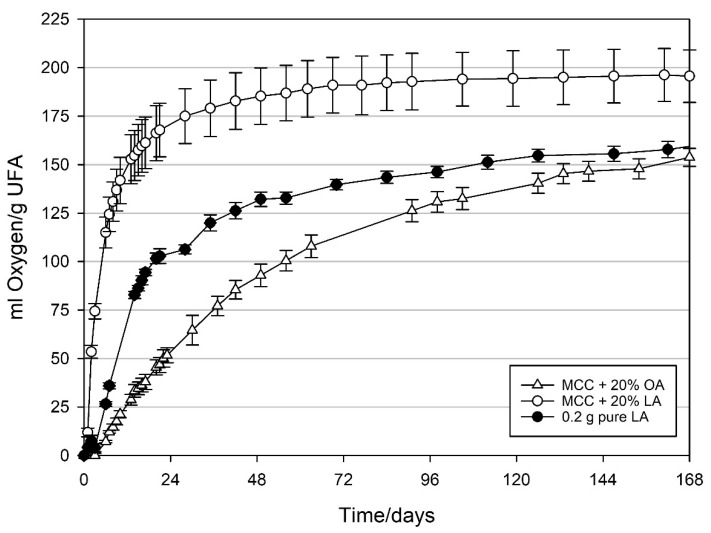
Oxygen absorption capacity of oleic and linoleic acid measured in packages containing 1 g modified calcium carbonate (MCC) loaded with 20 wt% oleic acid and linoleic acid, and 0.2 g pure linoleic acid. Packed under normal atmosphere (NA; headspace 250 mL), ~50% RH in the package and stored at 21 °C. Mean values and standard deviation (n = 4).

To determine the maximum oxygen absorption capacity of the MCC loaded with oleic and linoleic acid, they were packed at normal atmosphere (20.9 vol.-% O_2_) and oxygen concentration was monitored until depletion. As shown in Figure 9, maximum oxygen absorption capacity of MCC with UFAs was determined after six months as >195.6 ± 13.5 mL g_LA_^−1^ and >153.8 ± 4.7 mL g_OA_^−1^. For the pure LA, oxygen absorption capacity was >161.6 ± 3.4 mL g_LA_^−1^. The maximum absorption capacities are probably slightly higher than the given values due to the oxygen ingress into the packages during the storage time. However, the oxygen ingress at the experimental conditions (21 °C and 50% RH) is expected to be very low and below the standard deviations of the maximum oxygen absorption capacities.

### 3.4. Storage Stability of MCC Loaded with UFAs

**Figure 10:**
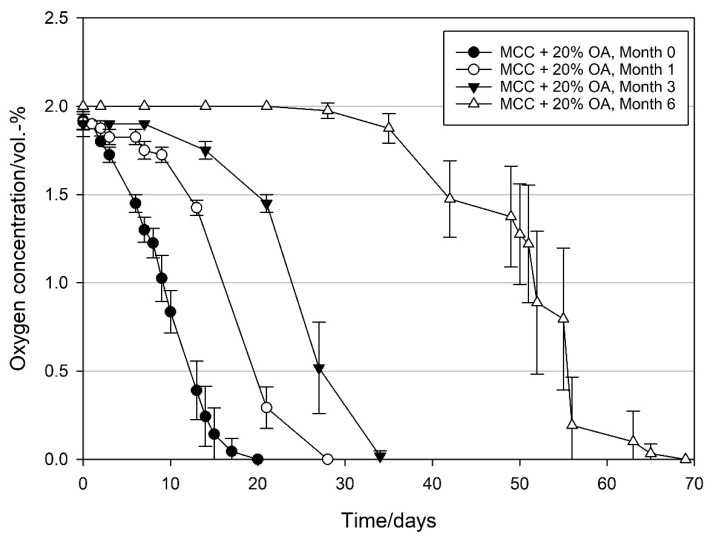
Oxygen scavenging concentration of modified calcium carbonate (MCC) loaded with 20 wt% oleic acid packed under modified atmosphere (MAP, 2 vol.-% O_2_, rest N_2_; headspace 250 mL), ~50% RH in the package and stored at 21 °C. Freshly produced oleic acid loaded MCC and same batch after one, three, and six months of storage at 21 °C under nitrogen and in the dark. Mean values and standard deviation (n = 4).

In order to evaluate how long the loaded MCC can be stored, MCC loaded with UFAs was stored under nitrogen in the dark at 21 °C, as described in Section 2.1, and their oxygen scavenging activity (OSA) was evaluated after one, three, and six months of storage. As shown in Figure 10, OSA of MCC loaded with OA declined during storage and the time required to remove the 2 vol.-% initial oxygen increased with increasing storage time from 20 to 28, 35, and 69 days after one, three, and six months, respectively. As depicted in Figure 10, the reduced activity is due to the prolongation of the initiation phase. The longer the storage time was the longer the initiation time was. Contrastingly, the average OSR (linear part of the slope) remained relatively constant with 1.7 ± 0.2, 1.7 ± 0.1, 1.9 ± 0.6, and 1.2 ± 0.2 mL O_2_ d^−1^ g_OA_^−1^ at day zero and one, three, and six months of storage, respectively.

**Figure 11:**
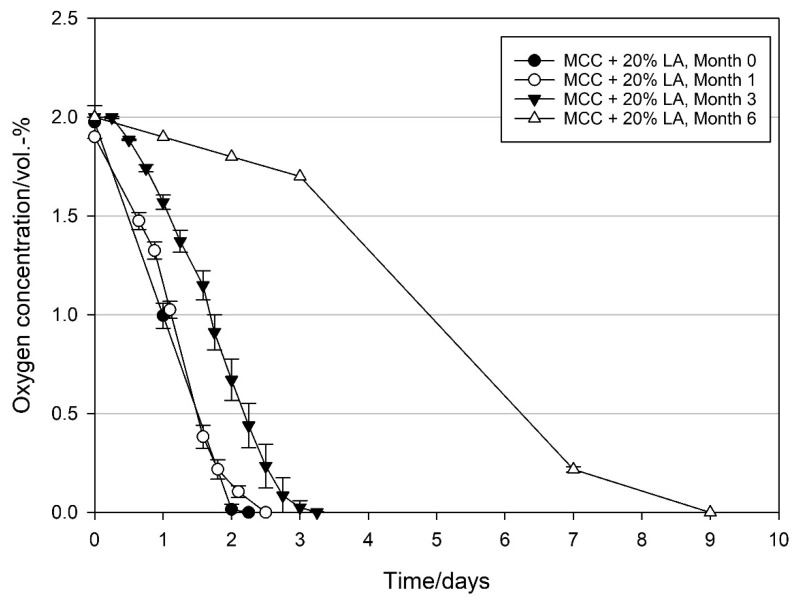
Oxygen scavenging concentration of modified calcium carbonate (MCC) loaded with 20 wt% linoleic acid packed under modified atmosphere (MAP, 2 vol.-% O_2_, rest N_2_; headspace 250 mL), ~50% RH in the package and stored at 21 °C. Freshly produced linoleic acid loaded MCC and same batch after one, three, and six months of storage at 21 °C under nitrogen and in the dark. Mean values and standard deviation (n = 4).

MCC loaded with LA showed a similar trend as OA loaded MCC, although it appears that the MCC loaded with LA is more stable in the first months (Figure 11). OSR also remained relatively stable within this timespan with 12.2 ± 0.6, 15.4 ± 0.7, and 11.1 ± 0.6 mL O_2_ d^−1^ g_LA_^−1^ at day zero and after one and three months of storage, respectively. After six months of storage, a clear decrease in oxygen scavenging activity was observed.

## 4. Discussion

So far, modified calcium carbonate (MCC) has been used as a carrier for release applications of pharmaceuticals [21,22]. More recently, MCC was uniquely investigated for the development of antimicrobial packaging demonstrating a controlled release of antimicrobial agents [23]. In the present study, the potential of MCC powder as carrier for unsaturated fatty acids (UFAs) to be used as oxygen scavengers has been shown. MCC was loaded with oleic acid (OA) and linoleic acid (LA), and oxygen scavenging activity (OSA) of the loaded MCC was evaluated under various conditions. 

Theoretically, oxygen uptake of fatty acids can follow three different types of reaction mechanisms namely β-oxidation, photosensitized oxidation, and autoxidation [2,39]. Within this study, it is our understanding that only autoxidation of oxygen takes place according to the reaction conditions and experimental setup. β-oxidation takes place in the presence of enzymes and is described in the fatty acid metabolism only. Photosensitized oxidation can also be excluded due to the lack of additional photosensitizes in the package and the experimental setup in the dark (package was stored in a climate chamber or fridge). Autoxidation of UFAs is described as a radical chain mechanism taking place at the double bonds of the fatty acid chain and can be divided into three phases: initiation, propagation, and termination. Thereby, both the duration of the initiation phase as well as the reaction rate in the propagation phase are dependent on the number of double bonds of the fatty acid chains [40,41]. Consequently, among all conditions tested (Section 3.2, Oxygen scavenging activity), different oxygen scavenging activities were observed for the applied fatty acids OA and LA, due to their given chemical structure. To initiate oxygen uptake, energy is required to remove hydrogen atoms adjacent to the double bond(s) [42]. Thereby, hydrogen atoms, which are located in between two double bonds, need lower energies for extraction. For this reason, the OSA of MCC loaded with LA, which has two double bonds (18:2), was remarkably higher compared to that of OA, which has only one double bond (18:1) and therefore a higher energy requirement for hydrogen extraction from the molecule. 

### 4.1. Effect of Loading Amount on the Oxygen Scavenging Activity of MCC Loaded with UFAs

MCC, the carrier material used in the present study, consists of partial recrystallized calcium carbonate containing variable layers of lamellae of calcium phosphate (hydroxyapatite) on the surface of the particles providing a remarkably high specific surface area of 103 m^2^ g^−1^, as visible on SEM images in Figure 2. In order to find the optimal loading amount of linoleic acid (LA) and oleic acid (OA) for oxygen scavenging, three different amounts of each UFA were loaded on MCC. As expected, an increase in the percent loading amount of both LA and OA led to increased filling of the available void space in the porous MCC. This was analytically confirmed by a reduction in intra particle specific pore volume and specific surface area, as shown in Table 1. However, as visible on SEM images, recorded from unloaded and UFA loaded MCC (Figure 2), the porous structure could be retained for all loadings as the pores were not fully filled.

With respect to oxygen scavenging activity (OSA) for both OA (Figure 3) and LA (Figure 4), a loading amount of 20 wt% was found to be the optimum, resulting in the highest oxygen scavenging rates (OSR) compared to the samples with higher and lower loadings of the respective UFA. Although more void space as well as a larger surface area was available at lower loadings of OA and LA (Table 1), the resulting OSA of the 20 wt% loadings indicate that with a 20 wt% UFA loading, where about half of the particle void spaces are filled, an optimal surface area/volume ratio is provided. In contrast, at the highest loadings, the OSA of both UFAs remarkably decreased, reflected by both strongly reduced surface area as well as pore volume (Table 1). It is therefore assumed that at loadings of 20 wt% and below, the UFAs are physically adsorbed on the MCC surface in the form of monolayers. Such monolayer arrangements are reported to build two-dimensional arrays of fatty acids at the interface between the substrate and the environment [43] which means in our case that the fatty acid chains are directly accessible for oxygen scavenging reaction at loadings of 20 wt% and below whereas the accessibility of the fatty acid chains is reduced at higher loadings.

Interestingly, in our study no oxygen scavenging activity was observed when pure OA was packed in the referring amount of the 20 wt% OA loading and stored under the same conditions. It is therefore assumed that the contact surface between oxygen and the 0.2 g pure liquid OA (~1 cm^2^) was insufficient to initiate autoxidation of OA. The observed increase in oxygen concentration in the package with pure OA can be attributed to the oxygen diffusion into the package, as depicted in Figure 3 (empty package). In packages containing pure LA, an oxygen uptake was observed, however, with an 8.7 times lower scavenging rate compared to that of MCC loaded LA in the referring amount (Figure 4). This difference is probably due to the low surface area (~1 cm^2^) of the 0.2 g pure liquid LA, on the glass petri dish, compared to the high surface area of the LA (20 wt%) distributed on porous MCC (0.527 cm^3^ g^−1^ intra particle pore volume and 31.6 m^2^ g^−1^ specific surface area after loading, Table 1). Furthermore, the diffusion of oxygen into the pores (filled with headspace gas) of MCC loaded LA is much faster than the diffusion in liquid LA which leads to a much faster reaction for MCC loaded LA than for pure liquid LA [2]. The observations made in the present study are therefore in line with previous studies describing that the extended surface area of MCC is ideal for higher oxidation kinetics [25,44]. In this context, it was shown by Pfaller [44] that MCC acted as a booster in the degradation of UFAs due to the spreading of the UFA over a large surface area, thus enabling oxygen exposure and action on a multitude of molecular sites. This is supported by the fact that the phenomenon is pronounced at lower loading levels and hence more exposed surface area. Similarly, the phenomenon is diminished at full loading where the pores are filled and oxygen access is strongly limited.

Comparison of OSA obtained in our study with the results of previous studies with UFAs is difficult due to the different test conditions as well as different carrier matrices used. Although many patent applications disclose UFAs as oxygen scavengers [29], scientific studies with UFAs as scavengers are scarce. Miranda [31] investigated the integration of oleic (18:1), linoleic (18:2), and linolenic (18:3) acid in poly(ethylene terephthalate) (PET) to improve its oxygen barrier properties. Among all UFAs tested, linoleic acid was found to have the highest potential as an oxygen scavenger due to its high utilization capacity (more details in Section 4.4 Oxygen absorption capacity), relatively fast kinetics, and lower cost. In a further study of Miranda [32], linoleic acid (0.5 wt%) was incorporated in PET bottles for oxygen barrier improvement and an oxygen scavenging rate of about 0.62 mL O_2_ d^−1^ cm^−2^ was achieved with the PET/LA bottles.

### 4.2. Effect of Temperature on the Oxygen Scavenging Activity of MCC Loaded with UFAs

Oxidation reaction mechanisms of UFAs are highly temperature dependent [2,39,45,46,47]. To evaluate the potential use of MCC loaded with UFA in food packaging, oxygen scavenging activity of MCC loaded with oleic acid and linoleic acid was evaluated at 5 °C complying with chilling room temperatures, 21 °C for ambient temperature and 30 °C for conditions with elevated temperatures. The results shown in Figure 5 and Figure 6 clearly demonstrate a temperature dependency of oxygen uptake with MCC loaded OA and LA, which is in correlation with previous experiments on oxidation of UFAs [2,45,46,47]. Furthermore, the observed prolongation of the initiation phase at decreasing temperatures correlates with autoxidation behaviour of fatty acids reported in literature [2,39]. Mainly the products stored at 5 °C are perishable products with high relative humidity and short shelf lives. Since the oxygen scavenging rates of MCC loaded with UFAs are very low at 5 °C and high relative humidity, such scavenging systems are likely to be unsuitable for the refrigerated food products. On the other hand, for dry products with low relative humidity and stored at room temperature, MCC loaded with UFAs, especially with LA, have high potential to be used as oxygen scavengers.

### 4.3. Effect of Relative Humidity on the Oxygen Scavenging Activity of MCC Loaded with UFAs

Apart from temperature, relative humidity has also been identified as a major factor to influence oxidation kinetics [48]. Depending on the water activity (a_w_) of a product that is packed, the relative humidity in the packaging headspace will be different [41]. The most common oxygen scavengers are based on iron and therefore have the serious disadvantage that their oxygen scavenging reaction does not progress in the absence of water since water is directly involved in the iron oxidation reaction [29]. Consequently, when dry products are packed, scavenger activation requires the addition of water. This water, however, may also migrate from the scavenger into the food, thereby negatively affecting the quality of the dry food [48].

In UFA oxidation, water is not directly involved in the reaction mechanism. Nevertheless, humidity influences their oxidation susceptibility in a complex way [41]. Thereby, different theories such as monolayer or glass transition theory on the effect of humidity on lipid oxidation are discussed controversially [49]. According to the monolayer theory, a monolayer of water covers the surface of the lipid, preventing it from direct exposure to oxygen. Since glass transition theory is more applicable to foods containing protein and/or saccharide polymers [50,51] monolayer theory could be more valid for the influence of relative humidity on the oxidation of UFAs. Generally, a decrease in the oxidation rates of UFAs with increasing humidity is reported [52]. This statement can be confirmed by the results obtained in the present study (Figure 7, OA and Figure 8, LA) clearly demonstrating the influence of RH on the oxygen scavenging activity of UFAs: The higher the RH, the lower the OSA. Particularly with increasing RH, a prolongation of the initiation phase was observed for both OA and LA. Thus, unsaturated fatty acids (UFAs) such as oleic or linoleic acid have been concluded to be suitable oxygen scavenging agents for the application of products with low or intermediary water activity. Based on the results obtained in the present study, water activity of products packed with an oleic acid-based oxygen scavenger are recommended to have an a_w_-value of <0.67, more preferably ≤0.47. For the application of a linoleic acid-based oxygen scavenger, a_w_-value of packed products is recommended to be ≤0.83, more preferably <0.67.

### 4.4. Oxygen Absorption Capacity of MCC Loaded with UFAs

The absorption capacity describes the maximum quantity of oxygen that can be absorbed by a scavenging agent [48]. This property must be known to design an oxygen scavenging packaging system where a specific amount of oxygen is aimed to be removed. The amount of oxygen that can be absorbed by unsaturated fatty acids can be estimated assuming that one mole of oxygen reacts with one mole of C=C. In the case of a 100% reaction turnover, one mole of oxygen would react with one mole of oleic acid (18:1) and two moles of oxygen with one mole of linoleic acid (18:2) [31].

For the UFAs loaded on MCC in the present study, a maximum oxygen absorption capacity of >195.6 ± 13.5 mL g_LA_^−1^ and >153.8 ± 4.7 mL g_OA_^−1^ as well as >161.6 ± 3.4 mL g_LA_^−1^ for pure LA was determined after six months. By investigating pure UFAs, Miranda [31] reported oxygen absorption capacities of 153 ± 5 mL g_LA_^−1^ and 10 ± 3 mL g_OA_^−1^. By adding a catalyst, they showed that the capacity of LA slightly decreased to 144 ± 5 mL g_LA_^−1^, whereas in the case of OA the capacity strongly increased to 70 ± 2 mL g_OA_^−1^. 

### 4.5. Storage Stability of MCC Loaded with UFAs

In practice, oxygen scavengers might be produced in large lot sizes and stored for some time after production and until their final application. For this reason, the storage stability of the UFAs loaded MCC was observed over a time span of six months of storage (under exclusion of oxygen). Whereas the average oxygen scavenging rates (linear part of the slope) remained relatively stable with the MCC loaded with oleic acid OA (Figure 10) or linoleic acid LA (Figure 11) over the whole storage period, the initiation phase of UFA oxidation was clearly prolonged during the storage time of six months.

The delayed initiation phase observed during the storage of the MCC loaded with OA and LA could be due to an adhesion (physisorption) [53] of nitrogen atoms present in the headspace of the storage container on the free reaction sites of the UFAs. The exchange of these nitrogen atoms with oxygen atoms in the packaging during the oxygen scavenging tests might have delayed the initiation of the oxidation. 

Although UFA-based oxygen scavengers exhibit excellent oxygen scavenging properties, to the authors’ knowledge, no commercial application has been implemented so far. A possible reason for this might be the main disadvantage that UFA oxidation comes along with odour-active by-products such as low-molecular-weight organic acids, aldehydes, or ketones, that occur during the reaction between the (poly-)unsaturated molecules and oxygen [29,30,41]. Migration of such substances from the scavenger into the package must therefore be prevented, since these molecules can adversely affect the sensory properties of packaged foodstuffs, even at very low concentrations, leading to consumer rejection or the raising of food regulatory issues. The application of functional barriers holding back organic compounds but providing oxygen permeability could be a possible approach [29]. Another way to minimize this problem could be the use of absorber/adsorber materials, particularly, if the UFAs are meant to be incorporated into polymer matrices.

## 5. Conclusions

In this study, it has been demonstrated that the highly porous modified calcium carbonate (MCC) provides a suitable carrier for integration of UFAs to be used as oxygen scavengers. MCC loaded with linoleic acid (LA) showed remarkably higher oxygen scavenging rates (12.2 ± 0.6 O_2_ d^−1^ g^−1^), and slightly higher oxygen absorption capacities (>195.6 ± 13.5 mL g^−1^), compared with MCC loaded with oleic acid (OA). For both LA and OA, oxygen scavenging activity decreased with increasing humidity (37–100% RH) and increased with rising temperatures (5–30 °C). Therefore, MCC loaded with UFAs would be particularly suitable for food products with low water activity and that are stored under non-refrigerated conditions. In a next step, MCC loaded with UFAs, most preferably linoleic acid, could be integrated into a packaging material to further study the oxygen scavenging performance under real packaging and storage conditions. In particular, food products with high unsaturated fatty acid content, such as fatty fish, plant-based oils, seeds or nuts, would be interesting to study the prevention of lipid oxidation. Additionally, it would be also interesting to study the potential of use of MCC as a carrier for other oxygen scavenging agents.

## Figures and Tables

**Table 1:** Intra particle pore volume and specific surface area of loaded and unloaded MCC. Mean values (n = 2).

Sample	Total Intra Particle Intruded Specific Pore Volume (0.004–0.34 μm) cm^−3^ g^−1^	Specific Surface Area m^2^ g^−1^
	1.019	103
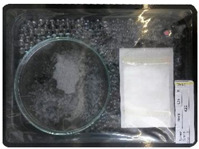 MCC + oleic acid		
9.1 wt% loading	0.802	72
20 wt% loading	0.648	49
41.5 wt% loading	0.163	6.6
MCC + linoleic acid		
10 wt% loading	0.766	60
20 wt% loading	0.527	31.6
30 wt% loading	0.352	8.9

## Data Availability

Not applicable.
